# Repeat kidney biopsy in patients with ANCA-associated vasculitis and suspected kidney relapse

**DOI:** 10.1093/ckj/sfag026

**Published:** 2026-02-05

**Authors:** Pierre Braud, Alice Le Clech, Grégoire Couvrat-Desvergnes, Jerôme Dimet, Anne Moreau, Karine Renaudin, Laurent Benard, Antoine Néel, Simon Ville, Charles Ronsin

**Affiliations:** Department of Nephrology and Immunology, University Hospital of Nantes, Nantes, France; Department of Nephrology, Saint Nazaire Hospital, Saint Nazaire, France; Department of Nephrology and Dialysis, Departmental Hospital of Vendée, La Roche-sur-Yon, France; Clinical Research Unit, Departmental Hospital of Vendée, La Roche-sur-Yon, France; Department of Pathology, Departmental Hospital of Vendée, La Roche-sur-Yon, France; Department of Pathology, University hospital of Nantes, Nantes, France; Department of Pathology, Saint Nazaire Hospital, Saint Nazaire, France; Department of Internal Medicine, University hospital of Nantes, Nantes, France; Department of Nephrology and Immunology, University Hospital of Nantes, Nantes, France; Centre de Recherche en Transplantation et Immunologie UMR 1064, Institut National de la Santé et de la Recherche Médicale (INSERM), Université de Nantes, Nantes, France; Department of Nephrology and Dialysis, Departmental Hospital of Vendée, La Roche-sur-Yon, France

**Keywords:** ANCA, biomarkers, kidney biopsy, proteinuria, vasculitis

## Abstract

**Background:**

The role of repeat kidney biopsy in antineutrophil cytoplasmic antibody (ANCA)-associated vasculitis (AAV) with suspected renal relapse remains uncertain. Clinical indicators such as hematuria, proteinuria and ANCA reappearance often guide therapy but may not reliably distinguish active vasculitis from chronic damage. Repeat biopsy may also provide prognostic information in patients with prolonged immunosuppressive exposure.

**Methods:**

We retrospectively analyzed adults with biopsy-proven AAV who underwent a second kidney biopsy (KB2) for suspected renal relapses. Clinical, laboratory and histopathologic data from the first (KB1) and KB2 were reviewed. Dynamic variations in ANCA, hematuria and proteinuria between 6 months post-KB1 and -KB2 were assessed for their association with histologic activity.

**Results:**

Forty patients were included; 25 (62%) had histologically active vasculitis on KB2, whereas 15 (38%) showed inactive lesions. Active disease was associated with the presence (*P* = .001) and higher levels of hematuria [100 (92–100) vs 14 (4–46) cells/mm³, *P* < .0001] and proteinuria [1.55 (1.0–3.30) vs 0.89 (0.44–1.53) g/g, *P* = .046]. Longitudinally, ANCA and hematuria reappearance were associated with active vasculitis in 68% and 80% of cases, respectively, whereas proteinuria rise was not. Repeat biopsies showed a shift from proliferative to sclerotic lesions, with increased glomerulosclerosis and interstitial fibrosis; the degree of glomerulosclerosis inversely correlated with renal recovery at 6 months.

**Conclusion:**

Clinical and laboratory findings alone often overestimate renal relapses. Dynamic changes in ANCA and hematuria improve prediction but remain insufficient to replace biopsy confirmation, which provides essential diagnostic and prognostic information in relapsing AAV.

## GRAPHICAL ABSTRACT

**GRAPHICAL ABSTRACT:**
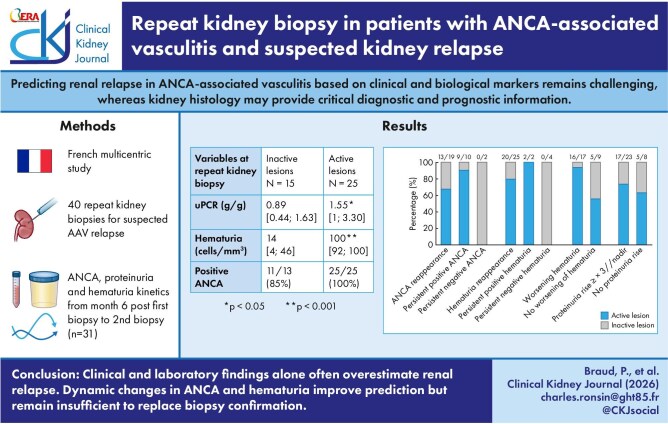

KEY LEARNING POINTS **What was known:**

- Clinical markers such as hematuria, proteinuria and antineutrophil cytoplasmic antibody (ANCA) are widely used to define renal relapses in ANCA-associated vasculitis (AAV) but their correlation with active vasculitis on repeat kidney biopsy has been rarely studied.
- The value of hematuria, proteinuria and ANCA dynamic changes to predict active renal vasculitis has not been studied.
- The prognostic contribution of repeat kidney biopsy in relapsing AAV had not been rigorously defined.

**This study adds:**

- Nearly 40% of clinically suspected renal relapses showed no histologic activity, demonstrating the limited accuracy of routine clinical and laboratory indicators.
- Dynamic changes in hematuria, but not proteinuria, were most closely associated with biopsy-proven renal activity.
- Repeat biopsy provides essential prognostic information through assessment of glomerulosclerosis and interstitial fibrosis.

**Potential impact:**

- Repeat kidney biopsy can prevent unnecessary immunosuppression in patients misclassified as relapsing based on clinical criteria alone.
- Histologic assessment refines prognostication and may guide treatment intensity in renal relapsing AAV.

## INTRODUCTION

Antineutrophil cytoplasm antibody (ANCA)-associated vasculitis (AAV) is a form of necrotizing vasculitis primarily targeting small vessels. It is characterized by the presence of autoantibodies directed against neutrophil proteins, either proteinase 3 (PR3) or myeloperoxidase (MPO). Renal involvement is a common manifestation of AAV, observed in approximately two-thirds of cases [1], which can lead to rapidly progressive glomerulonephritis and/or chronic kidney disease. Although initial treatment with immunosuppressive therapy often results in remission [2, 3], renal relapses are frequent, affecting approximately 40% of patients over a 5-year period [4]. This ongoing risk of relapse can threaten kidney function and compromise long-term prognosis [5, 6].

Renal biopsy is routinely performed at initial presentation in case of suspected renal involvement of AAV to confirm the diagnosis and to predict renal prognosis. However, its role in follow-up care in the context of suspected renal relapse is debatable. International guidelines offer limited clarity: the KDIGO 2024 [7] states that a “return or increase in hematuria with proteinuria may indicate a kidney relapse,” but does not address the clinical utility of repeat biopsy in this setting. In contrast, the European Alliance of Associations for Rheumatology (EULAR) 2024 [8] guidelines acknowledge that repeat kidney biopsy may help distinguish recurrent disease activity from chronic damage or alternative diagnoses. Although novel biomarkers have improved the ability to detect renal relapse in AAV [9–12], their limited availability restricts their use in routine practice, where assessment of suspected renal relapse still predominantly relies on serum creatinine, hematuria, proteinuria and ANCA dynamics. Immunosuppressive therapy is often initiated based on these clinical and laboratory findings alone, without histological confirmation of active renal vasculitis. Yet, these biomarkers are nonspecific and may fail to distinguish between active disease and inactive renal vasculitis [13–17], leading to potential misdiagnoses and inappropriate treatments.

As with the initial biopsy, repeat biopsy could offer important prognostic information, particularly regarding the extent of glomerular and interstitial fibrosis, which progressively accumulate over time [18–20] and influence renal outcomes. Evaluating fibrosis on repeat biopsy can guide therapeutic decisions, particularly in advanced chronic kidney disease where the risks of aggressive immunosuppression may outweigh its potential benefits.

In this study, we sought to assess the clinical utility of repeat kidney biopsies in AAV. Specifically, we conducted a multicenter retrospective analysis of patients who underwent a second biopsy for suspected renal relapse. Our objectives were to: (i) evaluate the diagnostic performance of routine plasma and urine biomarkers, particularly the dynamic changes in ANCA, hematuria and proteinuria, in identifying histologically confirmed active renal vasculitis; and (ii) characterize histopathological differences between initial and repeat biopsies and explore their associations with renal outcomes.

## MATERIALS AND METHODS

### Patients

Adult patients with AAV who underwent at least two kidney biopsies during their follow-up were retrospectively included from three centers in Western France from January 2000 to December 2024. Eligibility criteria required the following: (i) MPO- or PR3-positive ANCA, active renal disease in the first biopsy defined by a pauci-immune focal and segmental necrotizing and crescentic glomerulonephritis pattern and (ii) second kidney biopsy (KB2) performed because of suspected renal relapse. For patients with more than one follow-up kidney biopsy, only KB2 was included. Exclusion criteria included patients with eosinophilic granulomatosis with polyangiitis and other biopsy-proven kidney diseases not consistent with AAV glomerulonephritis at the first kidney biopsy. Data were collected retrospectively from electronic medical records. Characteristics at the first kidney biopsy (KB1) and KB2, histopathological parameters and Berden class were recorded.

### Definition

Active renal disease was defined histologically by fibrinoid necrosis, cellular and/or fibro-cellular crescent(s), erythrocyte tubular casts or the presence of glomerular fibrin on immunofluorescence [21]. Hematuria was defined as ≥10 red blood cells (RBC)/mm^3^ on cytological urine exam [22]. Acute kidney injury was defined according to the RIFLE (Risk, Injury, Failure, Loss, End-stage kidney disease) criteria [23] and alternatively, a rise in serum creatinine >30% consistent with the Birmingham Vasculitis Activity Score (BVAS) v3 criteria [22]. Estimated glomerular filtration rate (eGFR) was calculated according to Modification of Diet in Renal Disease (MDRD) formula [24]. eGFR in patients requiring dialysis was considered to be equal to 0. eGFR change at Month 6 (∆eGFR M0–M6) was calculated by subtracting eGFR at the time of KB2 (M0) from eGFR at Month 6 post-KB2 (M6). Vasculitis phenotype was classified according to the 2022 ACR/EULAR criteria [8].

Alternative diagnosis in patients with inactive vasculitis lesions on KB2 was established according to the conclusion of the medical report.

### Dynamic variation of ANCA, hematuria and proteinuria

At each immune-enzymatic determination of ANCA, information regarding ANCA status, hematuria count, urine protein-to-creatinine ratio (uPCR) and serum creatinine was collected from medical records spanning the remission period (Month 6 after induction treatment) to KB2. Repeated measurements of ANCA, hematuria and proteinuria were performed according to routine clinical follow-up and were not protocolized. ANCA detection methods were not standardized and included different commercially available assays. To determine the ANCA and hematuria profile, patients were longitudinally analyzed and classified according to ANCA and hematuria status between Month 6 post-KB1 and -KB2: (i) persistently negative (ANCA levels remained below the test’s lower detection range, RBC count remained below 10 cells/mm^3^, (ii) reappearance (ANCA became detectable or hematuria ≥10 RBC/mm^3^ after a prior negative result), and (iii) persistently positive (ANCA levels were consistently above the test’s lower detection range or hematuria was consistently >10 cells/mm^3^). In cases of fluctuating hematuria or ANCA status, such as transient reappearance of ANCA or hematuria that resolved in the subsequent sample, the patient was classified as having experienced a reappearance of hematuria or ANCA.

For hematuria determination, samples showing bacterial contamination were excluded to prevent confounding results from urinary infections. Due to inconsistent reporting of hematuria counts exceeding 100 RBC/mm³, values above this threshold were capped at 100 RBC/mm³. Two definitions of worsening hematuria were tested, the first was specified a priori as progression by at least one ordinal category of urinary RBC count: 0–49, 50–99 and ≥100 RBC/mm³; the second was a receiver-operating characteristic (ROC)-derived threshold determined on our cohort (reported as an exploratory, post-hoc cut-off).

Proteinuria “rise” was defined as a 3-fold rise (×3) from the nadir uPCR value between Month 6 post-KB1 and -KB2 (with a minimum value defined at 0.15 g/g, corresponding to the upper threshold for physiological proteinuria). The ×3 cutoff was chosen arbitrarily; however, alternative thresholds (×2, ×4, ×5) were also evaluated.

### Histological data

Kidney biopsy samples were reviewed at the time of the diagnosis by three renal pathologists (A.M., K.R., L.B.). Paraffin-embedded kidney sections stained with trichrome, silver, periodic acid–Schiff and hematoxylin/eosin.

Biopsy reports were reviewed retrospectively, and renal risk classification was determined according to the Berden score [25] (sclerotic class, ≥50% globally sclerotic glomeruli; focal class, ≥50% normal glomeruli; crescentic class, ≥50% cellular crescent and mixed class, <50% globally sclerotic, crescent and normal glomeruli) and ANCA renal risk score [26] for KB1 and KB2. Histopathological reports were systematically reviewed, and classification was based on the reported proportions of injured or normal glomeruli and the proportion of interstitial fibrosis and tubular atrophy (IFTA). Biopsies with <10 glomeruli were not excluded *a priori* in order to capture clinically meaningful cases and reflect real-world conditions in repeat biopsy settings. For the correlation analysis, % of crescentic lesions corresponded to % of cellular and fibro-cellular crescents per kidney biopsy and % of glomerulosclerosis referred to global glomerulosclerosis.

### Statistics and ethics

Continuous variables were summarized as median and interquartile range, and categorical and ordinal variables were summarized as frequencies and percentages. Comparisons between discrete variables were made using the Fisher’s exact test. For continuous variables, normality of continuous variables was assessed using the Shapiro–Wilk test, which showed that most variables were not normally distributed. Comparisons were made using an unpaired two-tailed Kruskal–Wallis test. For the correlation analysis, the Spearman test was used. ROC curve analyses were performed to evaluate the discriminatory ability of biomarkers. The area under the curve (AUC) was calculated, and statistical significance was assessed by testing whether the AUC differed from 0.5.

This study was exploratory in nature. The primary analyses were defined *a priori* and focused on the relationship between histologically active renal vasculitis at repeat kidney biopsy and dynamic changes in ANCA status and hematuria, based on biological plausibility and prior literature. These analyses were primarily descriptive and comparative. Secondary analyses, including alternative definitions of proteinuria rise and subgroup analyses, were considered exploratory and hypothesis-generating, hence formal adjustment for multiple testing was not applied. Missing data were handled using complete-case analysis and no imputation methods were applied. Statistical analyses were performed using R software version 4.4.0.

This study was approved by the Ethics Committee of the University Hospital of Nantes (number 24-91-07-222). Given the retrospective nature of the study, written informed consent was not required, but information letters about this study were provided to all patients included in the series.

## RESULTS

From January 2000 to December 2024, among the 44 patients who underwent repeat kidney biopsy, 40 fulfilled the inclusion criteria (four repeat kidney biopsies were excluded: three performed for refractory renal disease and one conducted systematically to assess renal scarring, Supplementary data, Fig. S1). Characteristics at KB1 are summarized in Supplementary data, Table S1. At the time of the first kidney biopsy, the median age was 66 (58–71) years. Ten patients (25%) had a granulomatosis with polyangiitis phenotype, and three (7.5%) presented with relapsing disease. The median BVAS v3 score was 13 (12–19), and the median serum creatinine level was 245 (176–348) µmol/L.

All but one patient received a maintenance immunosuppressive regimen after KB1, consisting of azathioprine in 11, mycophenolate mofetil in 10, rituximab in 12, steroid only in 4 patients, and 2 had switched azathioprine to mycophenolate mofetil or vice versa for toxicity. The median time between KB1 and KB2 was 4.3 (3; 7.8) years, and 29 (73%) patients had discontinued maintenance therapy at the time of KB2. The median time of follow-up after KB2 was 3.8 (1.6; 6.4) years.

### Clinical and biological parameters associated with active renal vasculitis at KB2

**Figure 1:**
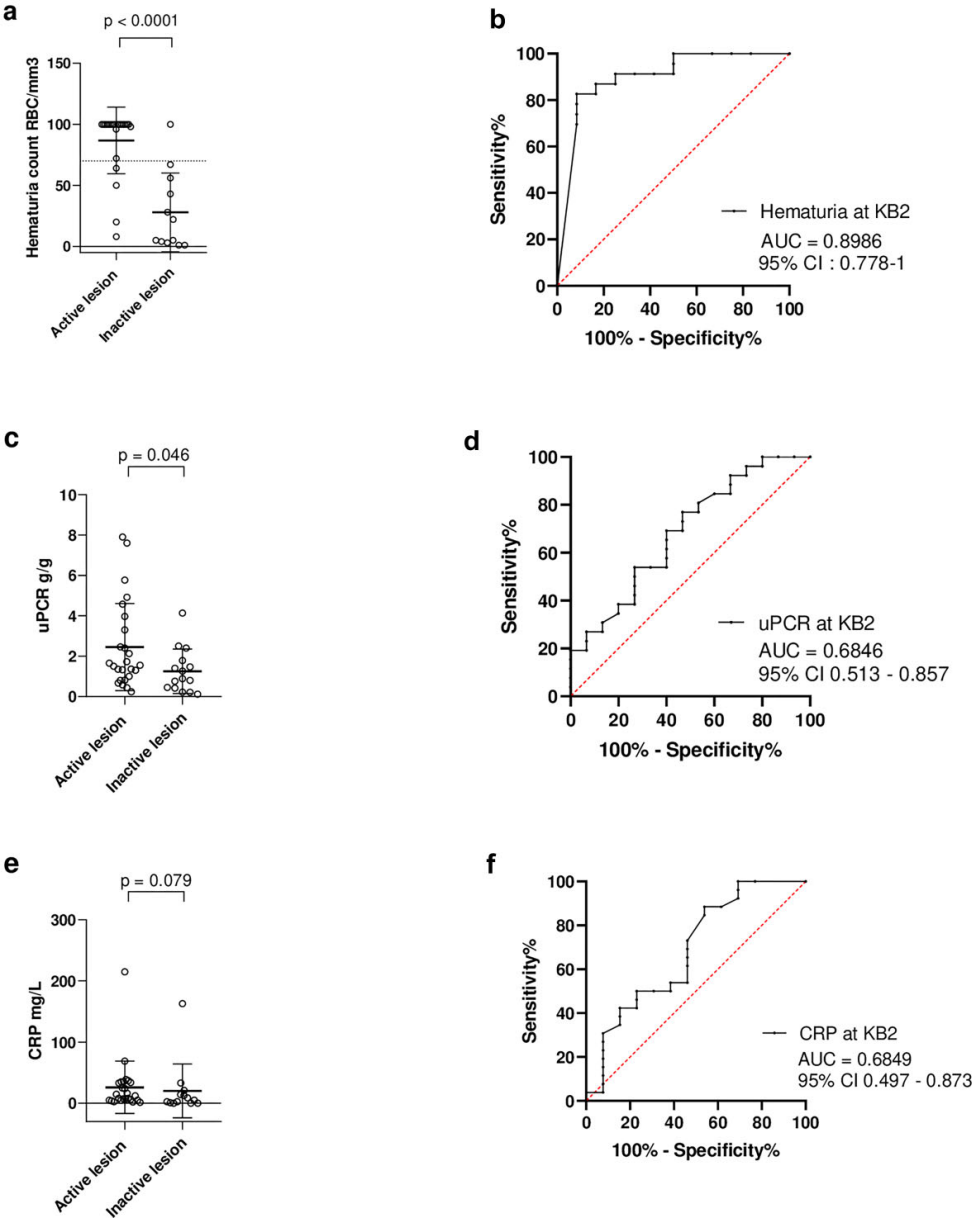
Evaluation of urinalysis and C-reactive protein testing according to the presence of active or inactive renal vasculitis at repeat kidney biopsy. (**a**) Comparison of hematuria count between patients with active or inactive renal vasculitis at repeat kidney biopsy. The dotted line reflects the optimal cut-off point of hematuria. Due to inconsistent reporting of hematuria counts exceeding 100 RBC/mm³, values above this threshold were capped at 100 RBC/mm³. (**b**) ROC curve for hematuria count comparing active with inactive vasculitis at repeat kidney biopsy. (**c**) Comparison of proteinuria between patients with active or inactive renal vasculitis at repeat kidney biopsy. (**d**) ROC curve for proteinuria comparing active with inactive vasculitis at repeat kidney biopsy. (**e**) Comparison of C-reactive protein between patients with active or inactive renal vasculitis at repeat kidney biopsy. (**f**) ROC curve for C-reactive protein comparing active with inactive vasculitis at repeat kidney biopsy.

Of the 40 patients with KB2 performed for suspected renal relapses, 25 had histologically proven active renal vasculitis and 15 had inactive renal vasculitis, resulting in a false clinical impression in 38% of cases. Factors associated with active renal vasculitis were the presence of hematuria (*P* = .001), hematuria count [100 (92; 100) vs 14 (4; 46), *P* < .0001], and higher proteinuria [1.55 (1; 3.30) vs 0.89 (0.44; 1.53), *P* = .046]. Conversely, the presence of glomerulosclerosis ≥50% at KB1 was associated with inactive renal vasculitis at KB2 (*P* = .036). Higher C-reactive protein (CRP) levels at KB2 were associated with a trend toward active renal vasculitis [12 (5–34) vs 5 (0.8–14) mg/L], although this difference did not reach statistical significance (*P* = .079). The ROC curve analysis indicated that proteinuria at the time of KB2 [AUC = 0.6846; 95% confidence interval (95% CI) 0.51; 0.86] and CRP (AUC = 0.6849; 95% CI 0.50; 0.87) poorly discriminated between active versus inactive vasculitis lesion. Conversely, hematuria count displayed better performance to discriminate between inactive versus active lesion (AUC 0.8986; 95% CI 0.78; 1). We used the ROC curve to define an optimum urinary RBC cut off ≥70 cells/mm^3^ to predict active renal vasculitis with a sensitivity of 83% (95% CI 63; 93) and a specificity of 92% (95% CI 65; 100) (Fig. 1).

Alternative diagnosis of the 15 kidney biopsies with inactive vasculitis disease reported in the medical report were drug toxicity for 4 patients (dehydration in patients with renin–angiotensin system inhibitor *n* = 3, proteinuria due to nintedanib *n* = 1), decompensated heart failure (*n* = 1), sepsis (*n* = 1), none reported or attributed to evolution of chronic kidney disease (*n* = 9).

All patients with active renal vasculitis at KB2 received a new induction immunosuppressive therapy regimen (Supplementary data, Table S2). Among patients with inactive renal vasculitis, one received immunosuppressive treatment for extra-renal disease. The remaining patient with extra-renal manifestation (peripheral neuropathy) did not receive additional immunosuppressive therapy.

At the end of follow-up [9 (7; 12) years from KB1], 14 patients (35%) had died: 7 from infectious disease, 3 from cardiovascular events, 1 from active vasculitis, 1 from malignancy, 1 from pulmonary embolism and 1 from an unknown cause.

### The dynamic of variation of ANCA, hematuria and proteinuria to predict renal vasculitis activity

We next wondered whether dynamic variation rather than static determination may predict histological vasculitis activity. Among the 40 patients included in the study, 31 had ≥3 ANCA, hematuria, proteinuria and serum creatinine determination between Months 6 post-KB1 and -KB2 and were included in this subgroup analysis. In this subgroup, 22 patients had active renal vasculitis at KB2, and 9 had inactive renal vasculitis at KB2. The median time between KB1 and KB2 was 4.2 (3.0; 7.8) years. The median ANCA, hematuria and proteinuria determinations were 9 (6; 12), 7 (5; 11) and 7 (5; 12), respectively, and the median time between each determination was 4.1 (2.8; 7.3) months.

### ANCA profile

**Figure 2:**
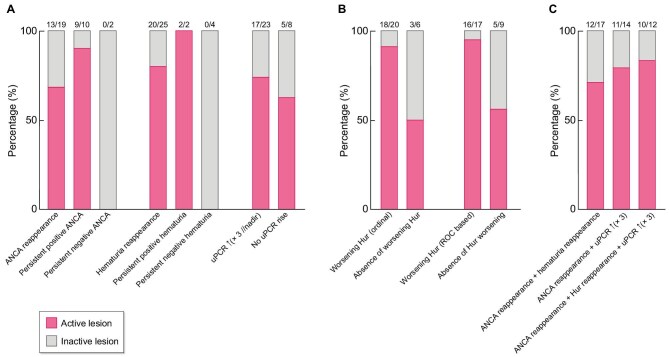
Dynamic change of ANCA, hematuria and proteinuria to predict histologically active renal vasculitis in AAV patients with repeat kidney biopsy for suspected renal relapse. (**a**) Percentage of active renal vasculitis on repeat kidney biopsy according to ANCA profile, hematuria profile and proteinuria rise (×3 increase from the nadir during the evolution between Month 6 post-KB1 to KB2). (**b**) Percentage of active renal vasculitis on repeat kidney biopsy in patients with or without worsening hematuria. This analysis included only patients with positive hematuria during M6 post-KB1 and -KB2. Ordinal definition: progression by at least one urinary RBC category (0–49, 50–99, ≥100 RBC/mm³). ROC-based definition: progression from <70 to ≥70 RBC/mm³. (**c**) Percentage of active vasculitis at KB2 when combining ANCA reappearance (ANCA reap), hematuria reappearance (Hur reap) and proteinuria rise (×3).

Of the 19 patients with ANCA reappearance (13 MPO ANCA, 6 PR3 ANCA), 13 (68%,10 MPO ANCA and 3 PR3 ANCA) had active vasculitis on KB2 (Fig. 2a) with a median time between ANCA reappearance and KB2 of 9.6 (2.1; 11.1) months. Among the six patients with ANCA reappearance and no active renal disease on KB2, two experienced subsequent disease relapses during follow-up: diffuse alveolar hemorrhage for one patient, 16.8 months after ANCA reappearance, and diffuse alveolar hemorrhage combined with rapidly progressive glomerulonephritis 26 months after ANCA reappearance for the other. Of the 10 patients with persistent ANCA, 9 (90%) had active renal vasculitis at KB2. The evolution of ANCA titers of these 9 patients is detailed in the Supplementary Results. Neither of the two patients with persistent negative ANCA had active renal vasculitis at KB2.

### Hematuria profile

Among the 25 patients with hematuria reappearance, 20 (80%) had biopsy-proven active vasculitis at KB2, with a median delay of 2.7 (1.1–5.9) months between hematuria onset and KB2 (Fig. 2a). Among the five patients without histological activity, one was receiving anticoagulation, another was premenopausal and three had no identifiable cause for hematuria. Of the 27 patients with positive hematuria during follow-up, hematuria count data were missing for one, leaving 26 patients for the analysis. When worsening hematuria was defined as progression by at least one ordinal category of urinary RBC count: 0–49, 50–99 and ≥100 RBC/mm³; 20 showed worsening hematuria, of whom 18 (90%) had active vasculitis. Among the six patients with positive hematuria but without worsening, three (50%) had active vasculitis at KB2 (*P* = .062). Using the ROC-derived threshold from Fig. 1 (worsening defined by progression from <70 to ≥70 RBC/mm^3^), 17 showed worsening hematuria, of whom 16 (94%) had active vasculitis. Among the nine with persistent but non-worsening hematuria (stable hematuria), five (56%) had active disease (*P* = .035) (Fig. 2b).

### Proteinuria profile

Among the 23 patients who had proteinuria rise (×3//nadir value), 17 (74%) had active renal vasculitis on KB2 (Fig. 2a). Among them, the median delay between proteinuria rise and KB2 was 1.3 (0.6; 7.3) months. Proteinuria rise (×3//nadir) was not associated with active renal vasculitis (*P* = .66). Other cut-offs for defining proteinuria rise were neither associated with active renal vasculitis (×2, ×4, ×5; Supplementary data, Fig. S2). Among the two patients who remained persistently ANCA-negative and underwent repeat kidney biopsy, both had a rise in proteinuria in the absence of hematuria; one also developed acute kidney injury. Neither showed evidence of active renal vasculitis on KB2.

### Temporal association between ANCA dynamics and urinary findings in patients with active renal vasculitis

Among patients with active vasculitis on KB2 showing both ANCA and hematuria reappearance (*n* = 12), the median interval between the two events was 8 (3–10) months, with ANCA reappearance preceding hematuria in nine cases and occurring simultaneously in three. Likewise, in those with both ANCA reappearance and a proteinuria rise (*n* = 11), the median delay between ANCA seroconversion and proteinuria rise was 10 (6–14) months; ANCA reappeared first in 10 cases and followed in 1.

### Sensitivity analysis according to biopsy adequacy

When restricting the analysis to repeat biopsies containing ≥10 glomeruli (*n* = 33), the association between hematuria and histologically active renal vasculitis remained consistent. In contrast, the associations with proteinuria (*P* = .053) and with glomerulosclerosis ≥50% (*P* = .071) were attenuated and did not reach statistical significance (Supplementary data, Table S3).

Similarly, when analyses of dynamic changes in ANCA, hematuria, and proteinuria were restricted to repeat biopsies containing ≥10 glomeruli on light microscopy (*n* = 25), the associations between biomarker dynamics and histologic activity remained consistent (Supplementary data, Fig. S3).

### Histological change between KB1 and KB2 and kidney outcomes according to KB2

#### Progressive increase in glomerular and interstitial fibrosis across the entire cohort

**Figure 3:**
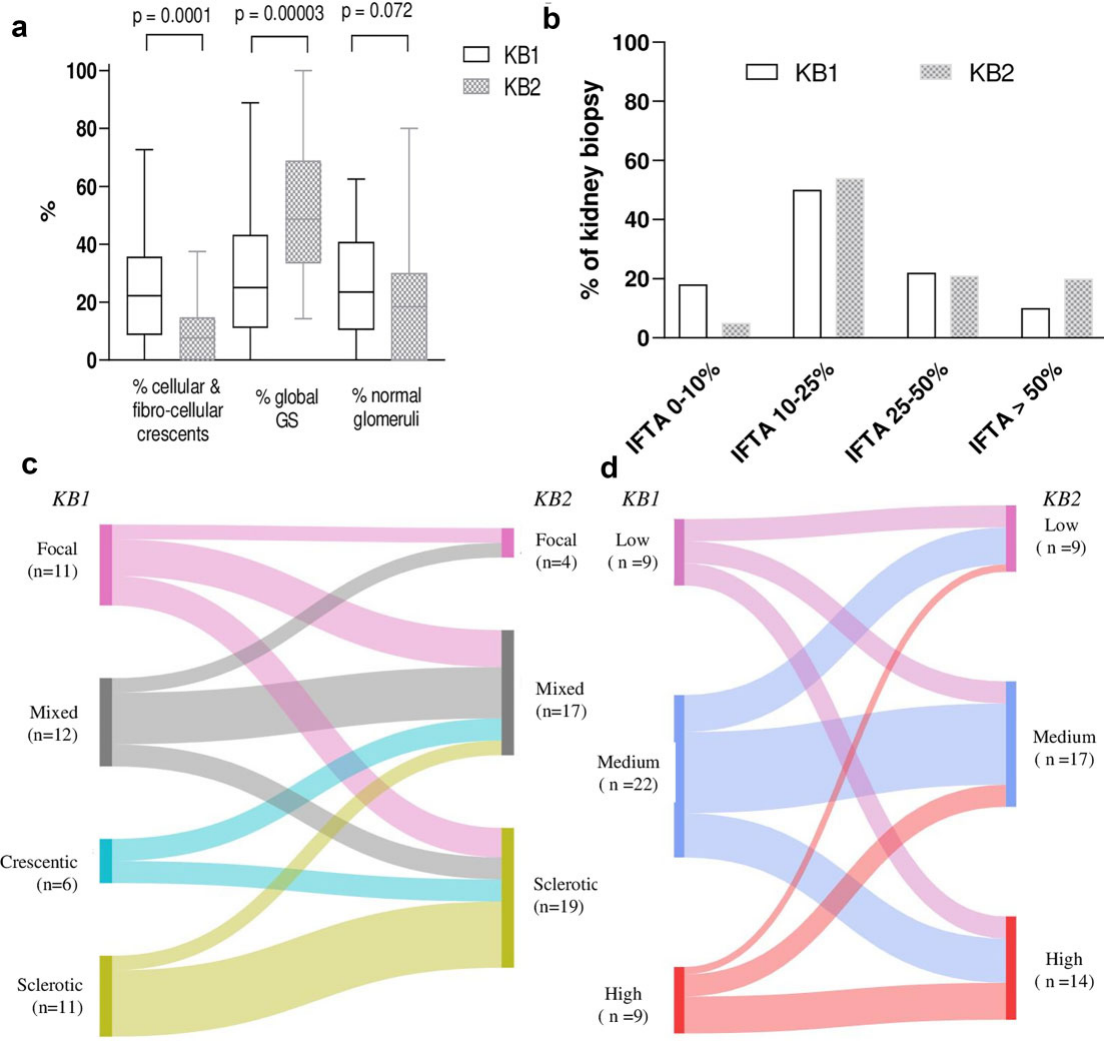
Histological change between first and second kidney biopsy in patients with AAV. (**a**) Percent of cellular or fibro-cellular crescent, global glomerulosclerosis and normal glomeruli according to KB1 or KB2. (**b**) Percent of IFTA according to KB1 or KB2. (**c**) Berden reclassification between KB1 and KB2. (**d**) ANCA renal risk score reclassification between KB1 and KB2. GS, glomerulosclerosis.

The median number of glomeruli per biopsy at KB1 was 15 (11; 25) and 14 (11; 24) at KB2. The first kidney biopsy showed a higher proportion of cellular or fibro-cellular crescents [22% (10; 36), *P* = .0001] and fewer glomerulosclerosis lesions [25% (11; 41) *P* = .00003]. Conversely, KB2 showed an increase in glomerulosclerosis lesions [49% (33; 68)] and a decrease in the proportion of cellular/fibro-cellular crescents [8% (0; 13)] (Fig. 3a). There was a trend toward a higher proportion of IFTA >50% at KB2 [8/40 (20%) versus 2/40 (5%), *P* = .087] (Fig. 3b) and fewer normal glomeruli [19% (2; 30) at KB2 vs 24% (12; 40) at KB1, *P* = .072]. At the first kidney biopsy, according to Berden’s classification, 11 patients (28%) had focal disease, 6 (15%) crescentic disease, 11 (28%) sclerotic disease and 12 (30%) mixed disease. The ANCA Renal Risk Score was low in 9 patients (23%), intermediate in 22 (55%) and high in 9 (23%).

Reclassification using Berden’s criteria between KB1 and KB2 showed a predominant sclerotic class in KB2 [19/40 (48%) versus 11/40 (28%) at KB1, *P* = .11], although without reaching statistical significance and a decreased proportion of crescentic class [0/40 (0%) at KB2 versus 7/40 (18%), *P* = .011] (Fig. 3c). Reclassification using the ANCA Renal Risk Score between KB1 and KB2 showed a non–statistically significant increase in the proportion of patients in higher risk categories [9 patients (23%) at KB1 versus 14 (35%) at KB2; *P* = .32] (Fig. 3d). Reclassification of Berden classes and ANCA renal risk scores between KB1 and KB2 did not differ according to ANCA specificity (MPO versus PR3, Supplementary data, Fig. S4). No significant association was observed between induction or maintenance immunosuppressive therapies at KB1 and the degree of glomerulosclerosis or interstitial fibrosis/tubular atrophy at KB2 (Supplementary data, Tables S4 and S5).

#### In patients with active vasculitis, glomerular scarring at repeat biopsy is associated with poor renal recovery

**Figure 4:**
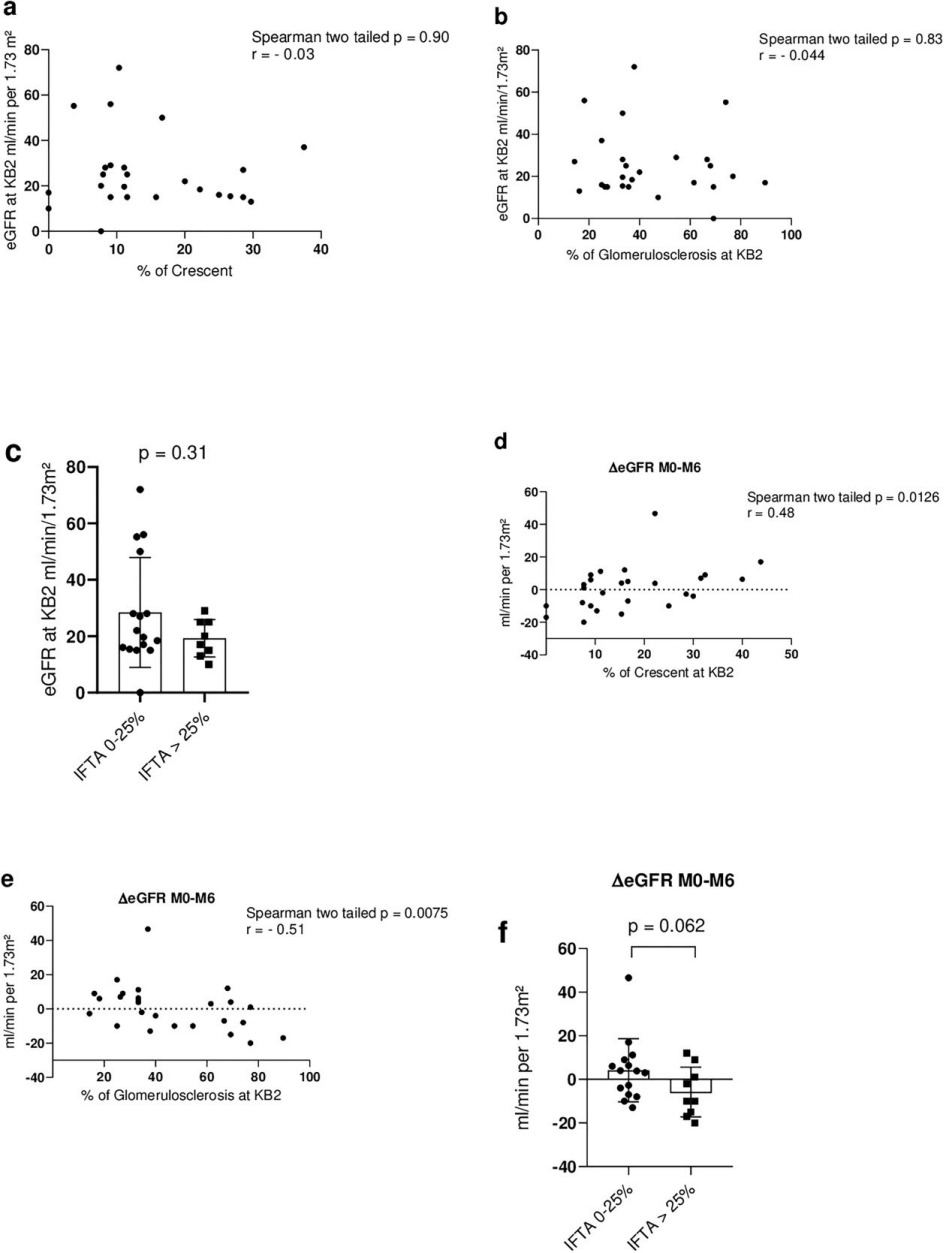
Histological changes at KB2 in the 25 patients with active renal vasculitis and renal outcomes. (**a**–**c**) Correlation between % of crescent, % of global glomerulosclerosis and IFTA at KB2 and eGFR at KB2. (**d**–**f**) Correlation between % crescent, % of global glomerulosclerosis and IFTA at KB2 and eGFR changes (∆eGFR M0–M6) at Month 6 post-KB2. ∆eGFR M0–M6 was calculated by subtracting the eGFR at KB2 by the eGFR at Month 6 post-KB2, one patient had missing data at Month 6 post-KB2 and was excluded from the analysis. For the correlation analysis, Spearman test was used with two tailed *P*-value. eGFR is by MDRD.

Among the 25 patients with active vasculitis at KB2, the percentage neither of crescent lesions, glomerulosclerosis nor IFTA correlated with eGFR at KB2 (Fig. 4a–c). However, the eGFR change between KB2 and Month 6 post-KB2 (∆eGFR M0–M6) was inversely correlated with the percentage of glomerulosclerosis (*P* = .075, r = –0.51). Conversely, the percentage of cellular or fibro-cellular crescentic lesion positively correlated with eGFR change 6 months post-KB2 (*P* = .0126, r = 0.48) (Fig. 4d and e). IFTA >25% showed a trend toward less eGFR increase between M0 and M6 [+4.0 (–3.7; +8.3) for IFTA 0%–25% and –10 (–15; 1) for IFTA > 25% *P* = .062] (Fig. 4f).

## DISCUSSION

In this multicenter study, we evaluated the diagnostic accuracy of routine biomarkers and the role of repeat kidney biopsy in patients with AAV and suspected renal relapse. Three main findings emerged. First, nearly 40% of clinically suspected relapses were not confirmed histologically, underscoring the limitations of relying solely on clinical and laboratory parameters. Second, longitudinal changes in ANCA and hematuria may suggest active renal vasculitis, although it is not fully specific. Third, repeat biopsies consistently revealed progression of chronic scarring, with prognostic implications for post-treatment renal recovery. Our findings complement recent reports on rfepeat kidney biopsy in AAV [15, 16] by providing additional data on the longitudinal dynamics of routinely available biomarkers between biopsies and their relationship with histologic activity. Unlike prior studies, we specifically examined temporal changes in ANCA and urinary findings between the first and repeat biopsy, an aspect that has been only sparsely explored to date.

Consistent with previous reports, hematuria and proteinuria are not specific for active renal vasculitis [13, 14] and may instead reflect chronic scarring or alternative etiologies. Serial monitoring of hematuria may be more informative: new onset hematuria was associated with histologically active renal vasculitis in 80% of cases, and worsening hematuria was associated with active renal vasculitis. Previous studies reported similar associations, although without biopsy-proven vasculitis [27]. Conversely, when a renal relapse is suspected, the absence of hematuria makes the presence of histologically active renal vasculitis unlikely.

While proteinuria is a well-established of marker of kidney damage [28], renewed attention has been directed toward its predictive value for renal relapses [29, 30] and its presumed correlation with ongoing glomerular inflammation [30]. In our study, proteinuria at KB2 was a weak biomarker to predict histologically renal vasculitis, and increases over time was not associated with histological activity. This may be explained by fluctuations in proteinuria among patients with extensive glomerulosclerosis, which could confound clinical assessment and contribute to the higher prevalence of histologically inactive lesions at KB2 in cases where glomerulosclerosis exceeded 50% at KB1.

In our cohort, over two-thirds of patients exhibiting ANCA reappearance had biopsy-proven active vasculitis. Additionally, a subset of patients with histologically inactive vasculitis at KB2 subsequently experienced renal and/or extra-renal relapse within several months. Consistent with prior reports, ANCA reappearance was associated with systemic disease recurrence [31]. Notably, in patients with active renal vasculitis at KB2, asymptomatic serological reactivation frequently preceded hematuria by several months, suggesting a smoldering resurgence of systemic autoimmunity. Similar concepts of “serological activity” preceding clinical relapse have been described in other glomerulopathies [32–34] and may also apply in AAV given the role of ANCA in its pathogenesis [35], although this remains speculative and warrants further study.

As expected, repeat biopsies showed a clear shift from active proliferative lesions to chronic scarring, with increased glomerulosclerosis and interstitial fibrosis. This pattern has been described in other series and reflects the cumulative burden of kidney injury over time [15, 18–20]. Importantly, our study demonstrated that the degree of glomerulosclerosis on the second biopsy was inversely associated with renal function recovery after immunosuppressive therapy. These results support the prognostic utility of repeat biopsy in distinguishing patients who may still benefit from aggressive immunosuppression from those in whom extensive fibrosis indicates limited potential for recovery.

Our findings highlight the need for an integrated approach combining serial biomarker monitoring with targeted use of repeat biopsy. While dynamic changes in ANCA and hematuria can guide suspicion of relapse, histologic confirmation remains crucial before initiating potentially toxic immunosuppressive therapy. Repeat biopsy also provides prognostic information by documenting the degree of irreversible scarring, which should be factored into therapeutic decision-making. Such an approach may optimize the balance between treatment efficacy and safety in this high-risk population. Yet, kidney biopsy remains limited by its invasive nature and the possibility of sampling error, particularly in focal vasculitis or in kidneys with advanced glomerulosclerosis. Albeit non-specific to AAV [8], urinary CD163 and CD4+ T cells have emerged as promising biomarkers for renal vasculitis [9, 12]. Notably, urinary CD163 correlates with glomerular inflammation at the time of AAV diagnosis [10]. However further research is needed, particularly regarding the temporal evolution of urinary CD163 and CD4 T cells before histological evidence of active renal vasculitis.

Apart from its limited sample size, our study has several limitations. The timing of ANCA measurement, urine analysis, and serum creatinine assessment varied between patients, which restricts the timeline analysis of the relationships between ANCA reappearance, hematuria recurrence, and proteinuria rise. Heterogeneity in maintenance regimens, variability in the interval between biopsies and the long study period during which major changes in AAV treatment occurred may have influenced histologic and clinical outcomes. The absence of data on antiproteinuric treatment prescription at each proteinuria point may have affected proteinuria level. Although ANCA titers may fluctuate in association with extrarenal disease activity, this potential confounding was partly mitigated as no patients with documented extrarenal relapses between the first and second kidney biopsies were included. Nevertheless, subclinical extrarenal activity cannot be entirely excluded. Finally, given the exploratory design and the limited sample size, our findings should be interpreted as hypothesis-generating and require confirmation in larger prospective cohorts.

In conclusion, dynamic changes in ANCA and hematuria improve prediction of histologic activity but remain insufficient as stand-alone indicators. Repeat biopsy should therefore be considered in selected cases to refine therapeutic strategies and to optimize the risk–benefit balance in the management of relapsing AAV (Table 1).

**Table 1:** Factors associated with active histological vasculitis on repeat-KB performed for suspected renal relapses.

Variables	Whole sample, *n* = 40	Missing (not available)	Inactive lesion on KB2, *n* = 15	Active lesion on KB2, *n* = 25	*P*
At KB1					
% of normal glomeruli	24 (12; 40)	2	18 (14; 28)	29 (9; 43)	.52
Glomerulosclerosis ≥50%	8/40 (20)	0	6/15 (40)	2/25 (8)	**.036**
IFTA 0–10%	4/38 (11)	2	4/15 (27)	3/23 (13)	.40
IFTA ≥25%	13/38 (34)	2	3/15 (20)	10/23 (43)	.18
Proteinuria at Month 6 post-KB1 ≥0.5 g/g	21/33 (64)	7	10/12 (83)	11/21 (52)	.13
At KB2					
Age (years)	72 (66; 76)	0	73 (57; 78)	72 (68; 77)	.86
Female	20/40 (50)	0	9/15 (60)	11/25 (44)	.51
Median time between KB1 and KB2 (years)	4.3 (3;7.8)	0	4.4 (3.4; 7.7)	4.2 (2.8; 7.8)	.46
Maintenance immunosuppressive treatment at the time of KB2	6/40 (15)	0	2/15 (13)	4/25 (16)	1
Extra renal involvement, *n* (%)	8/40 (20)	0	2/15 (13)^a^	6/25 (24)	.69
Serum creatinine (µmol/L)	233 (200; 283)	0	222 (186; 261)	243(210; 289)	.42
Acute kidney injury^b^	28/40 (70)	0	9/15 (60)	19/25 (76)	.31
Rise in serum creatinine >30%^c^	22/40 (55)	0	7/15 (47)	15/25 (60)	.52
uPCR (g/g)	1.38 (0.8; 2.4)	0	0.89 (0.44; 1.63)	1.55 (1; 3.30)	**.046**
Hematuria ≥10 cells/mm^3^	29/37 (78)	3	6/13 (46)	23/24 (96)^d^	**.001**
Hematuria count (cells/mm^3^)^e^	97 (24; 100)	5	14 (4; 46)	100 (92; 100)	**<.0001**
Positive ANCA MPO or PR3	36/38 (95)	2	11/13 (85)	25/25 (100)	.11
White blood cells (G/L)	7.2 (6.0;9.1)	0	6.7 (6.4; 7.9)	7.4 (6.0; 9.4)	.56
Hemoglobin (g/dL)	11.1 (9.9; 12.1)	0	11.5 (10.3; 12.1)	11 (9.7; 12)	.47
Platelet (G/L)	274 (211; 316)	0	250 (217; 292)	283 (187; 321)	.74
C-reactive protein (mg/L)	9 (4; 31)	2	5 (0.8; 14)	12 (5; 34)	.079

Continuous variables are expressed in median (interquartile range) and categorical and ordinal variables are expressed as frequencies and percentages. Comparisons between discrete variables were made using the Fisher’s exact test. For continuous variables, comparisons were made using an unpaired two-tailed Kruskal–Wallis test.

“Two tailed Kruskal-Wallis test.” “Bold values indicate statistical significance (p < 0.05).”

^a^ANCA reappearance, arthralgia, epistaxis and persistence of proteinuria around 1.4 g/g (without proteinuria rise) and C-reactive protein <5 mg/L for one patient and ANCA reappearance, hematuria reappearance without acute kidney injury or proteinuria rise, peripheral neuropathy and C-reactive protein at 33 mg/L.

^b^RIFLE criteria.

^c^BVAS criteria.

^d^One patient had hematuria reappearance 12 months before KB2 (18 RBC/mm^3^), 1 month after hematuria reappearance, hematuria subsequently disappeared.

^e^Due to inconsistent reporting of hematuria counts exceeding 100 RBC/mm³, values above this threshold were capped at 100 RBC/mm³.

## Supplementary Material

sfag026_Supplemental_File

## Data Availability

The dataset used and analysed in the current study are available from the corresponding author upon reasonable request.
