# Supplementary material for: Repeat kidney biopsy in patients with ANCA-associated vasculitis and suspected kidney relapse
Source: Clin Kidney J. 2026 Feb 5;19(3):sfag026. doi: 10.1093/ckj/sfag026 (PMC13148155; doi:10.1093/ckj/sfag026)

Supplemental material

Supplemental result

Persistent positive ANCA.

Among the nine patients with active renal vasculitis and persistent ANCA, titer evolution (measured using the same immune-enzymatic assay) showed an increase in one patient, decrease in 1 patient, ANCA titer above the maximal value. For 3 patients ANCA titer assay was not determined by the same immunoenzymatic assay.

Supplemental Table S1. Baseline characteristics at the first kidney biopsy and treatment of the 40 patients.

**Baseline characteristics and treatment**	**Whole sample at 1st KB (n =40)**
**At diagnosis**	
Age (years)	66 [58;71]
Female	20/40 (50)
ANCA anti MPO	31/40 (78)
ANCA anti PR3	9/40 (22)
Microscopic polyangiitis	30/40 (75)
Granulomatosis with polyangiitis	10/40 (25)
BVASv3 score	13 [12;18]
Extra renal involvement	19/40 (48)
Constitutional symptoms	10/40 (25)
Musculoskeletal	5/40 (13)
Cutaneous	3/40 (8)
Purpura	2/40 (5)
Gangrene	1/40 (3)
Pulmonary	12/40 (30)
Alveolar hemorrhage	7/40 (18)
Interstitial lung disease	4/40 (10)
Pleural effusion	1/40 (3)
Nodules	1/40 (3)
ENT	7/40 (18)
Crusting rhinitis	4/40 (10)
Sinusitis	2/40 (5)
Otitis media	2/40 (5)
Neurological	1/40 (3)
Peripheral neuropathy	1/40 (3)
Cardiac	1/40 (3)
Pericarditis	1/40 (3)
Laboratory data	
Serum creatinine µmol/L	245 [176;348]
uPCR g/g	1.8 [1;2.6]
Hematuria	34/36 (94)
C-reactive protein mg/L	17 [6;93]
Need for dialysis at diagnosis	5/40 (13)
**Induction regimen**	40/40 (100)

Steroids	40/40 (100)
Cyclophosphamide	23/40 (58)
Rituximab	12/40 (30)
Mycophenolate mofetil	3/40 (8)
Plasma exchange	4/40 (10)
**Maintenance regimen**	39/40 (98)
Steroids only	4/40 (10)
Azathioprine	11/40 (28)
Rituximab	12/40 (30)
Mycophenolate mofetil	10/40 (25)
Azathioprine/ mycophenolate mofetil switch	2/40 (5)
**ACEi or SGLT2i treatment between KB1 and KB2[a]**	20/40 (50)
Within the first 6 months following KB1	15/40 (38)
After month 6 post KB1	5/40 (12)

Continuous variables are expressed in median [inter-quartile range] and categorical and ordinal variables are expressed as frequencies and percentages. Abbreviation. ACEi, angiotensin converting enzyme inhibitor and SGLT2i, sodium glucose transport 2 inhibitor. MPO, myeloperoxidase; PR3, proteinase 3; uPCR, urinary protein to creatinine ratio.

Supplemental Table S2. Treatment and outcomes of the 25 patients with active renal vasculitis at KB2.

**Variables**	**Active renal vasculitis at KB2 n =25**
**Induction regimen**	N = 25
Corticosteroid only	3/25 (12)
Rituximab	16/25 (64)
Mycophenolate mofetil	5/25 (20)
Cyclophosphamide	1/25 (4)
Avacopan	3/25 (12)
**ACEi or SGLT2i treatment**	15/25 (60)
**Outcomes at month 6**	N = 24[a]
eGFR (ml/min/1.73m²)	22 [19;33]
eGFR gain (ΔeGFR M0-M6)	3 [-7;8]
End stage renal disease	5/24 (21)
**Outcomes at last follow up**	N= 25
Median time of follow-up after KB2 (years)	3.9 [0.9;8.3]
Relapses	4/25 (16)
eGFR (ml/min/1.73m²)	21 [0;26]
End stage renal disease	11/25 (44)
Median time between KB2 and ESRD (years)	2 [0.3;5]
Death	10/25 (40)

Continuous variables are expressed in median [inter-quartile range] and categorical and ordinal variables are expressed as frequencies and percentages. Patients requiring dialysis or who received renal transplant were considered to have an eGFR at 0ml/min/1.73m².

[a] one patient had missing data at month 6. eGFR, estimated glomerular filtration (MDRD); ESRD, end stage renal disease. ACEi, angiotensin converting enzyme inhibitor or Sodium glucose transport 2 inhibitor.

Supplemental Table S3. Factors associated with active histological vasculitis on repeat-kidney biopsy with at least 10 glomeruli performed for suspected renal relapses.

Variables	Whole sample n = 33	Missing (not available)	Inactive lesion on KB2 n = 12	Active lesion on KB2 n = 21	p
**At KB1**					
% of normal glomeruli	24 [13 ;41]	1	15 [13 ;25]	29 [17 ;46]	0.16
Glomerulosclerosis ≥50%	7/33 (21)	0	5/12 (42)	2/21 (10)	0.071
IFTA 0-10%	6/33 (18)	0	3/12 (25)	3/21 (14)	0.64
IFTA ≥ 25%	12/33 (36)	0	3/12 (25)	9/21 (43)	0.46
Proteinuria at month 6 post KB1 ≥0.5g/g	17/28 (61)	5	8/10 (80)	9/18 (50)	0.23
**At KB2**					
Age (years)	71 [60 ;75]	0	69 [50 ;75]	72 [68 ;75]	0.37
Female,	17/33 (52)	0	7/12 (58)	10/21 (48)	0.72
Median time between KB1 and KB2 (years)	4.4 [3.1;7.8]	0	4.3 [3.6 ;6.3]	4.6 [2.8 ;7.8]	0.74
Maintenance immunosuppressive treatment at the time of KB2	5/33 (15)	0	2/12 (17)	3/21 (14)	1
Extra renal involvement, n (%)	6/33 (18)	0	1/12 (8)[a]	5/21 (24)	0.38
Serum creatinine µmol/L	237 [189 ;304]	0	228 [176 ;286]	247 [207 ;304]	0.59
Acute kidney injury[b]	23/33 (70)	0	7/12 (58)	16/21 (76)	0.43
Rise in serum creatinine > 30%[c]	17/33 (52)	0	5/12 (42)	12/21 (57)	0.48
uPCR (g/g)	1.4 [0.8 ;2.4]	0	1.08 [0.37 ;1.55]	1.55 [1.3 ;3.3]	0.052
Hematuria ≥10C/mm3	24/31 (77)	2	4/10 (40)	20/21 (95)[d]	**0.002**
Hematuria count (c/mm3)[e]	99 [27 ;100]	5	5 [3 ;28]	100 [97 ;100]	**0.0004**
Positive ANCA MPO or PR3	30/32 (94)	1	9/11 (82)	21/21 (100)	0.11
White blood cells (G/L)	7.4 [6;9]	0	7.4 [6.4 ;8.5]	7 [6 ;9.4]	0.90
Hemoglobin (g/dL)	11.3 [9.8 ;12.2]	0	11.5 [9.8 ;11.98]	11.1 [9.7 ;12.2]	0.67
Platelet (G/L)	278 [207 ;317]	0	260 [216 ;298]	283 [187 ;321]	0.90
C-reactive protein (mg/L)	8 [3.3 ;28.5]	2	5.8 [0.2 ;13.73]	8 [5 ;34]	0.15

Continuous variables are expressed in median [inter-quartile range] and categorical and ordinal variables are expressed as frequencies and percentages. Comparisons between discrete variables were made using the Fisher's exact test. For continuous variables, comparisons were made using an unpaired 2-tailed Kruskal-Wallis test.

[a] ANCA reappearance, arthralgia, epistaxis and persistence of proteinuria around 1.4g/g (without proteinuria rise) and CRP < 5mg/L. [b] RIFLE criteria.[c] BVASv3 criteria.[d] One patient had hematuria reappearance 12 months before KB2 (18 red blood cells/mm3), one month after hematuria reappearance, hematuria subsequently disappeared. [e] Due to inconsistent reporting of hematuria counts exceeding 100 RBC/mm³, values above this threshold were capped at 100 RBC/mm³.

Supplemental Table S4. Glomerulosclerosis at repeat kidney biopsy according to treatment at baseline.

	Total N = 40	Glomerulosclerosis <50% at KB2 N = 21	Glomerulosclerosis ≥ 50% at KB2 N= 19	p
**Induction regimen at KB1**				
Cs + Cyclophosphamide	23/40 (58)	13/21 (62)	10/19 (53)	0.75

Cs + Rituximab	12/40 (30)	7/21 (33)	5/19 (26)	0.73
Cs + MMF	3/40 (8)	0/21 (0)	3/19 (16)	0.10
Cs only	2/40 (5)	1/21 (5)	1/19 (5)	1
**Maintenance regimen between KB1 and KB2**				
Rituximab	12/40 (30)	5/21 (24)	7/19 (37)	0.49
Azathioprine or MMF	23/40 (58)	12/21 (57)	11/19 (58)	1
Corticosteroids only	4/40 (10)	3/21 (14)	1/19 (5)	0.61
None	1/40 (3)	1/21 (5)	0/19 (0)	1
**ACEi or SGLT2i at KB1**	20/40 (50)	10/21 (48)	10/19 (53)	1

ACEi, angiotensin converting enzyme inhibitor and SGLT2i, sodium glucose transport 2 inhibitor. MMF, mycophenolate mofetil; Cs, corticosteroid. Comparisons between discrete variables were made using the Fisher's exact test.

Supplemental Table S5. Interstitial fibrosis and tubular atrophy at repeat kidney biopsy according to treatment at baseline.

	**Total N = 40**	**IFTA < 50% at KB2 N = 32**	**IFTA≥ 50 % at KB2 N = 8**	**p**
**Induction regimen at KB1**				
Cs + Cyclophosphamide	23/40 (58)	20/32 (63)	3/8 (38)	0.25
Cs + Rituximab	12/40 (30)	8/32 (25)	4/8 (50)	0.21
Cs + MMF	3/40 (8)	2/32 (6)	1/8 (13)	0.50
Cs only	2/40 (5)	2/32 (6)	0/8 (0)	1
**Maintenance regimen between KB1 and KB2**				
Rituximab	12/40 (30)	9/32 (28)	3/8 (38)	0.67
Azathioprine or MMF	23/40 (58)	19/32 (59)	4/8 (50)	0.70
Corticosteroids only	4/40 (10)	3/32 (9)	1/8 (13)	1
None	1/40 (3)	1/32 (3)	0/8 (0)	1
**ACEi or SGLT2i at KB1**	20/40 (50)	15/32 (47)	5/8 (63)	0.69

ACEi, angiotensin converting enzyme inhibitor and SGLT2i, sodium glucose transport 2 inhibitor. MMF, mycophenolate mofetil; Cs, corticosteroid. Comparisons between discrete variables were made using the Fisher's exact test.

Supplemental Figure.

Supplemental Figure S1. Flow chart of the study.

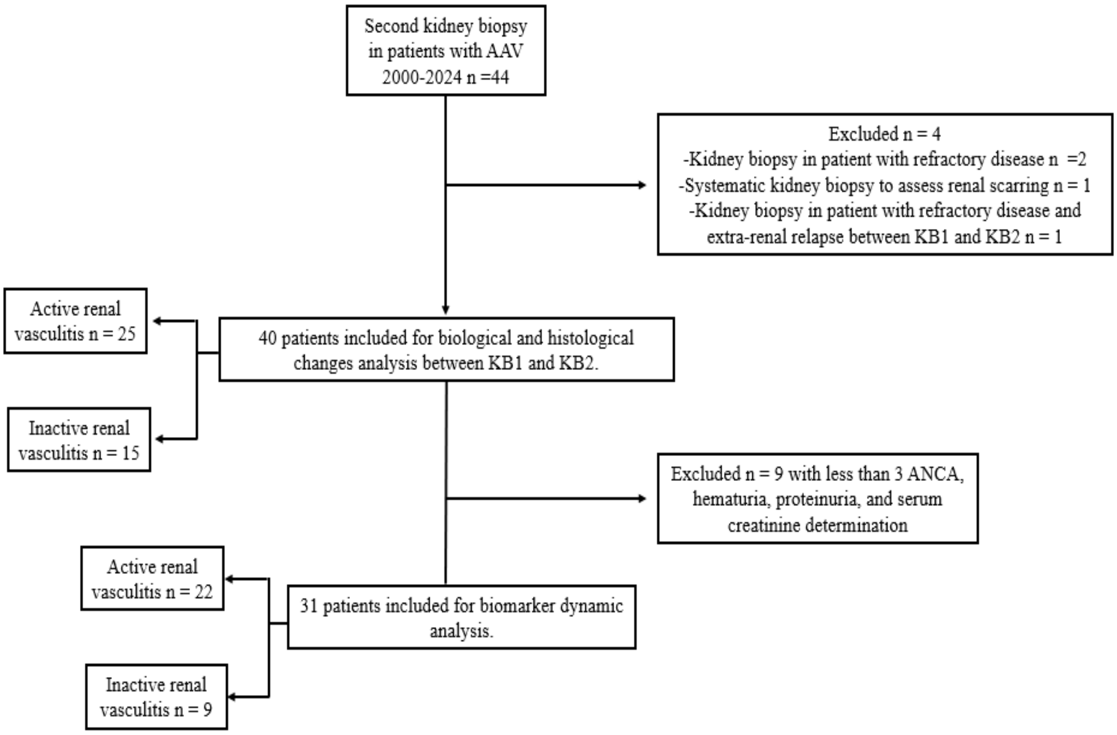

Supplemental Figure S2. Urinary to protein/creatinine rise and active vasculitis on KB2 according to proteinuria rise definition.

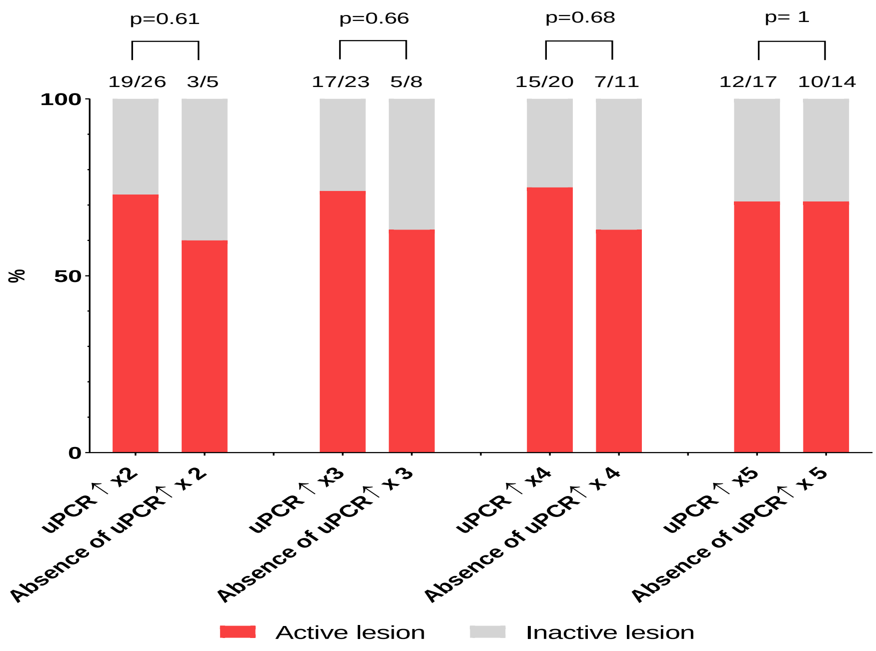

Supplemental Figure S3. Dynamic changes in ANCA, hematuria, and proteinuria predicting histologically active renal vasculitis in patients with ANCA-associated vasculitis undergoing repeat kidney biopsy for suspected renal relapse with ≥10 glomeruli in repeat biopsy sample. **a**) Percentage of active renal vasculitis on repeat KB according to ANCA profile, hematuria profile and proteinuria rise (x3 increase from the nadir during the evolution between month 6 post KB1 to KB2). **b**) Percentage of active renal vasculitis on repeat KB in patients with or without worsening hematuria. This analysis included only patients with positive hematuria during M6 post KB1 and KB2. Ordinal definition: progression by at least one urinary red blood cell category (0–49, 50–99, ≥100 RBC/mm³). ROC-based definition: progression from <70 to ≥70 RBC/mm³ **c**) Percentage of active vasculitis at KB2 when combining ANCA reappearance (ANCA reap), hematuria reappearance (Hur reap) and

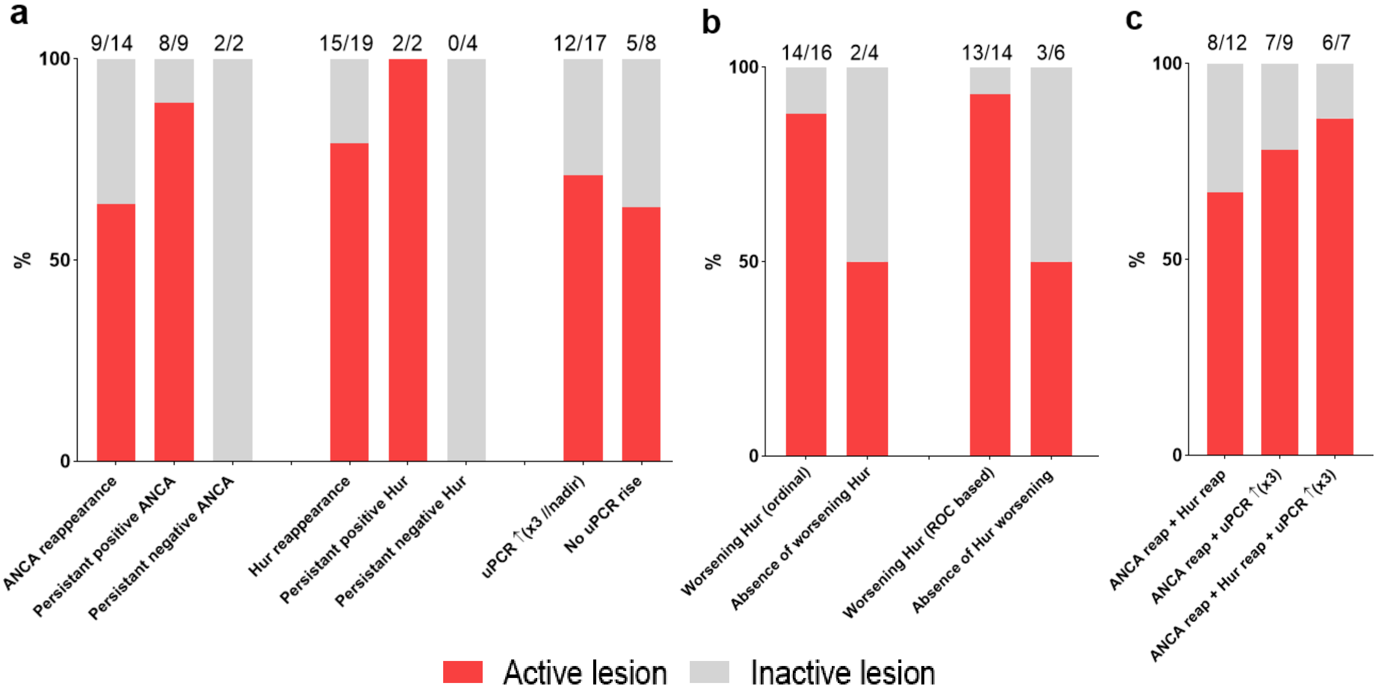

Supplemental Figure S4. Berden and ANCA renal risk score reclassification between the first and second kidney biopsy according to ANCA status. **a**) Berden reclassification. **b**) ANCA renal risk score reclassification.

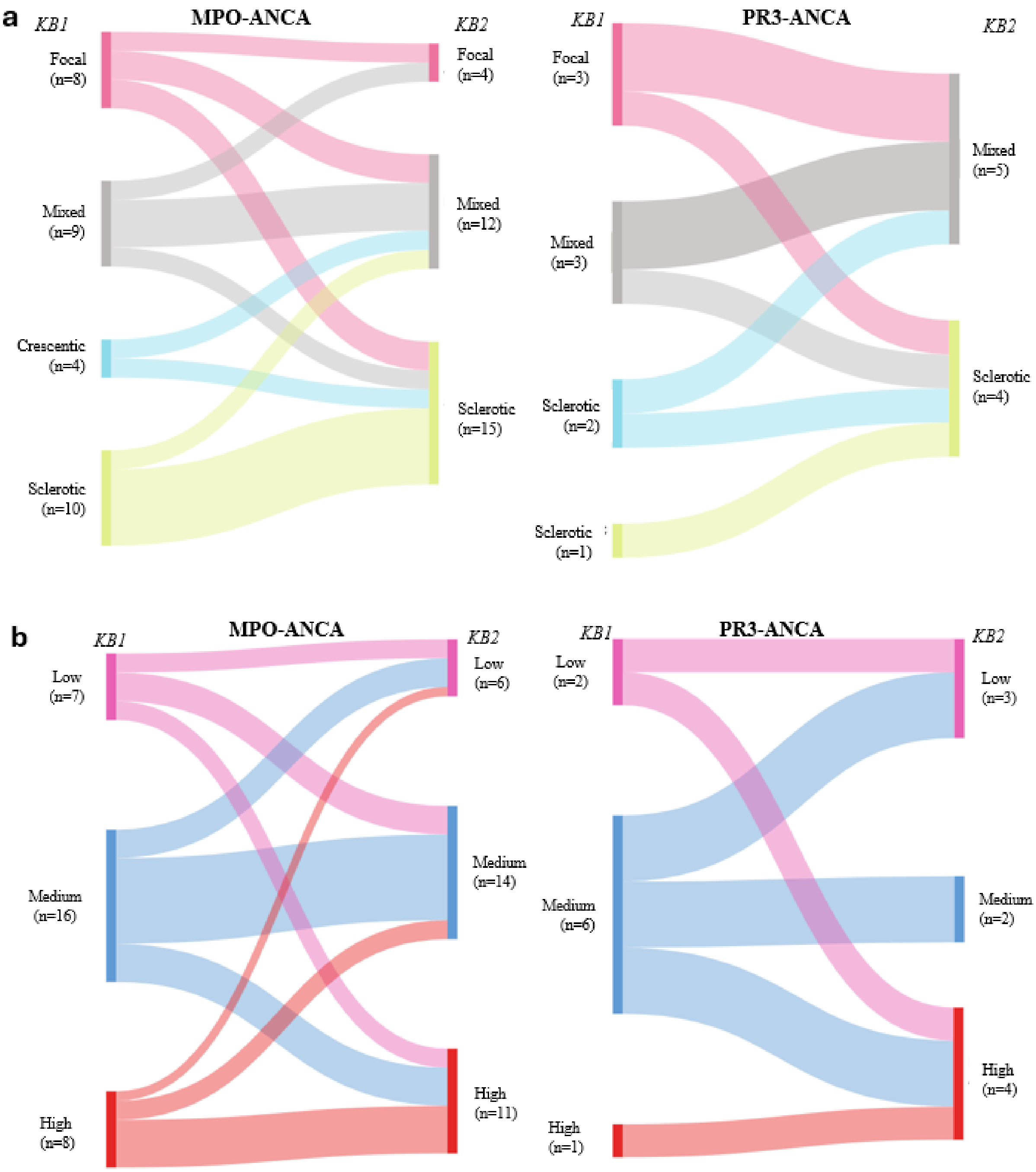

Supplement: sfag026_Supplemental_File [file sfag026_supplemental_file.pdf]
